# Is value-based choice repetition susceptible to medial frontal transcranial direct current stimulation (tDCS)? A preregistered study

**DOI:** 10.3758/s13415-021-00889-7

**Published:** 2021-04-02

**Authors:** Ulrike Senftleben, Johanna Kruse, Franziska M. Korb, Stefan Goetz, Stefan Scherbaum

**Affiliations:** 1 Department of Psychology, Technische Universität Dresden, Dresden, Germany; 2 Pratt School of Engineering, Duke University, Durham, NC 27708 USA; 3 School of Technology, University of Cambridge, Cambridge, CB2 1RX UK; 4 School of Medicine, Duke University, Durham, NC 27710 USA

**Keywords:** Decision making, Choice repetition, Transcranial direct current stimulation, Medial prefrontal cortex

## Abstract

In value-based decision making, people have to weigh different options based on their subjective value. This process, however, also is influenced by choice biases, such as choice repetition: in a series of choices, people are more likely to repeat their decision than to switch to a different choice. Previously, it was shown that transcranial direct current stimulation (tDCS) can affect such choice biases. We applied tDCS over the medial prefrontal cortex to investigate whether tDCS can alter choice repetition in value-based decision making. In a preregistered study, we applied anodal, cathodal, and sham tDCS stimulation to 52 participants. While we found robust choice repetition effects, we did not find support for an effect of tDCS stimulation. We discuss these findings within the larger scope of the tDCS literature and highlight the potential roles of interindividual variability and current density strength.

**Supplementary Information:**

The online version contains supplementary material available at 10.3758/s13415-021-00889-7.

## Introduction

When we make decisions, we are not only influenced by the decisions we face right now, but also by our previous decisions. Our previous decisions—our decision history—impact our decision making both on a short-term timescale (i.e., the current decision affects the next decision) and on a longer timescale (i.e., the current decision affects the next couple of decisions). Such so-called sequential effects have been studied extensively in the context of perceptual decision making (Cho et al., 2002; Fründ et al., 2014; Gao et al., 2009; Soetens et al., 1985, 2004; Urai et al., 2019). Recently, we showed that similar effects also play a role in value-based decision making in the form of choice repetition—a sequential effect characterized by the tendency to repeat a decision in the subsequent trial instead of switching to an alternate decision —or hysteresis (Scherbaum et al., 2016; Schoemann & Scherbaum, 2020; Senftleben et al., 2019). Thus, the underlying mechanisms of sequential effects seem to affect decision making in a variety of domains. But what exactly are these mechanisms?

Several studies have used biologically plausible computational models to show that choice repetition emerges from the residual activity from the previous trial (Bonaiuto et al., 2016; Gao et al., 2009; Rustichini & Padoa-Schioppa, 2015; Scherbaum et al., 2016; Senftleben et al., 2019). This residual activity creates an advantage for the previously chosen option, which in turn makes it more likely that this option will be selected again in the new trial and leads to faster decision times for repetitions.

**Fig. 1.**
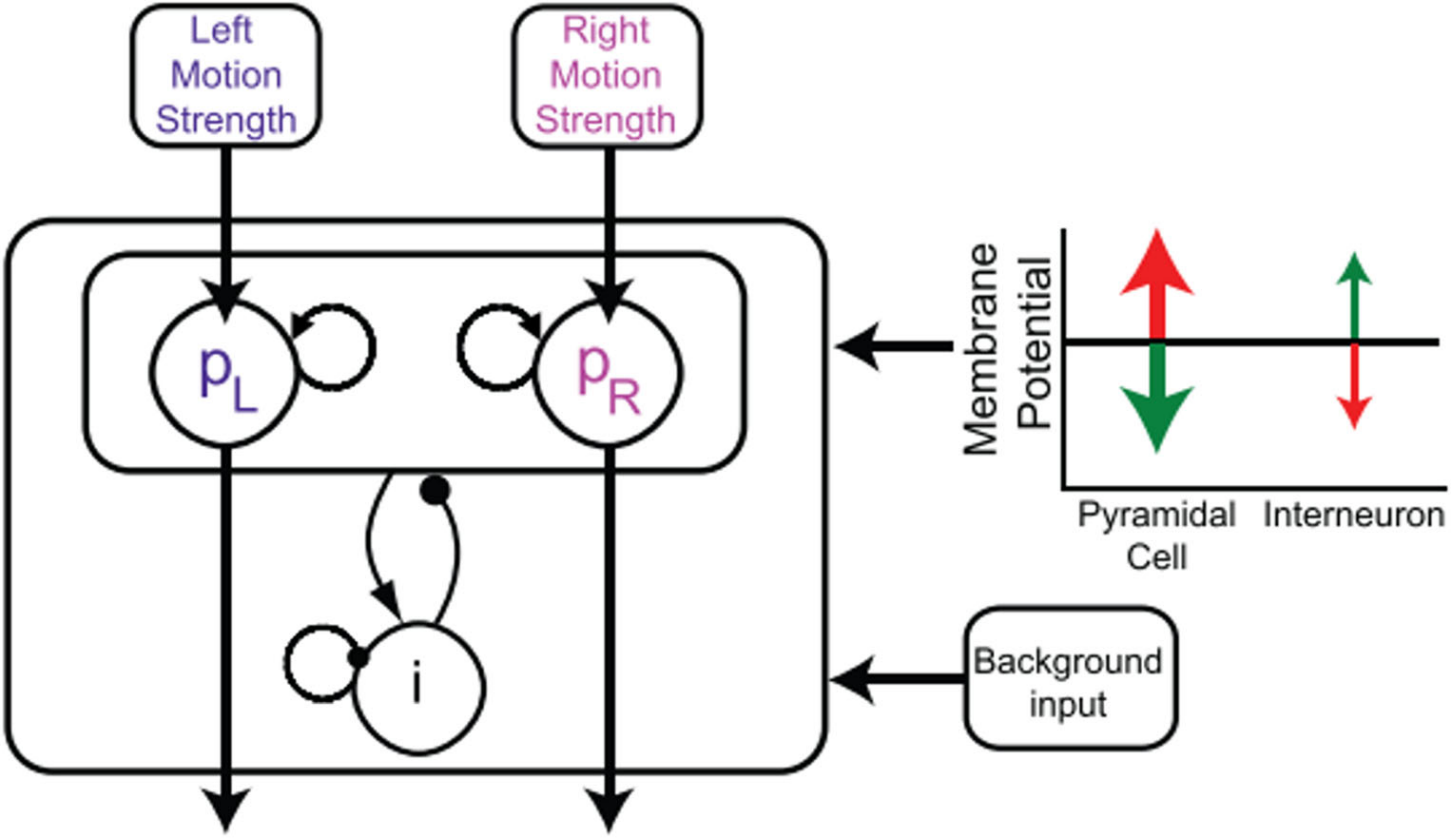
Computational model by Bonaiuto et al. (2016). The model simulates decision making in a random dot kinematogram task, where the task is to identify coherent motion to the left or the right. The model consists of two populations of pyramidal cells (p_L_ and p_R_) and a common pool of inhibitory interneurons (i). The pyramidal cell populations receive the task-related evidence as input (left and right motion strength). Each pyramidal cell population possesses self-excitation, and they mutually inhibit each other through the interneurons. In addition, the model includes background noise. The influence of transcranial direct current stimulation is simulated via changes of the membrane potential of pyramidal cells and interneurons. Anodal stimulation (red) leads to a depolarization of pyramidal cells, whereas cathodal stimulation (green) leads to a hyperpolarization of pyramidal cells. Figure adapted from Bonaiuto et al. (2016), DOI: 10.7554/eLife.20047.003.

Recently, Bonaiuto et al. (2016) probed this account by altering the decay of residual activity through transcranial direct current stimulation (tDCS) in a perceptual decision task. Transcranial direct current stimulation is a brain stimulation method where a small electric current is sent through the brain by placing two electrodes on the scalp of participants. This electric current changes network dynamics by de- or hyperpolarizing the resting membrane potential (Nitsche et al., 2008; Nitsche & Paulus, 2011). Bonaiuto et al. (2016) modeled the effect of tDCS stimulation on perceptual choice repetition using a biologically plausible neural network model. In their model, two populations of neurons sample evidence for two decision options (see Fig. 1 for an illustration of the model architecture). Each population boosts its own activity through self-excitation. The two populations are connected through inhibitory interneurons, meaning that the activity of one population suppresses activity in the other population. Through these processes of self-excitation and mutual inhibition, a winner-takes-all dynamic emerges and the model settles into a stable state (either for option 1 or option 2), which marks the decision. After the model made a decision, the simulated neural activity decays over time, slowly returning to a neutral resting level. If a new decision trial starts before both neural populations have returned to resting level, this influences the new decision and creates a repetition bias. Thereby, Bonaiuto and colleagues’ computational modeling analysis revealed that choice repetition emerges naturally from their biophysical attractor model, which is in line with other neural network models of decision making (Berlemont & Nadal, 2019; Cho et al., 2002; Gao et al., 2009; Rustichini & Padoa-Schioppa, 2015; Scherbaum et al., 2016; Senftleben et al., 2019). Crucially, the authors further showed that tDCS can alter choice repetition by changing the membrane potential. Specifically, they modeled the influence of tDCS through depolarization (anodal tDCS) and hyperpolarization (cathodal tDCS) of the membrane potential. This affected how fast activity from the previous trial decayed after a decision had been made. Under depolarization, it took longer for the residual activity of the previous trial to decay. Therefore, when the next decision trial started, this residual activity biased the decision toward repeating the previous decision. Through this mechanism, depolarization amplified choice repetition effects and led to faster decision times. In contrast, hyperpolarization led to a faster decay of residual activity and therefore decreased choice repetition effects and increased decision times. The authors tested these model predictions empirically through applying tDCS over the left dorsolateral prefrontal cortex (dlPFC) during a perceptual decision making task. As predicted, choice repetition was stronger under depolarizing (anodal) stimulation and weaker under hyperpolarizing (cathodal) stimulation, which strongly supports the notion that choice repetition is caused by the residual activity from the previous trial. In addition, decision times were faster under depolarizing and slower under hyperpolarizing stimulation, as predicted by the model.

Recently, we used a similar model to successfully predict choice repetition effects in value-based decision making (Scherbaum et al., 2016; Schoemann & Scherbaum, 2020; Senftleben et al., 2019). In value-based decision making, it is assumed that people make decisions based on subjective values that they attribute to the different choice options. This value signal is generated in the medial prefrontal cortex (mPFC), the brain region most strongly associated with value-based decision making, which has been demonstrated in a large number of neuroimaging studies (Hare et al., 2009; Hunt et al., 2012; Kable & Glimcher, 2007; Rolls et al., 2008; Rushworth et al., 2011), physiological animal studies (Padoa-Schioppa & Assad, 2006; Strait et al., 2014; Wallis & Miller, 2003), as well as lesion studies (Camille et al., 2011; Fellows, 2006; Gläscher et al., 2012). On a behavioral level, we have shown in previous studies that humans show value-based choice repetition effects (Scherbaum et al., 2016; Schoemann & Scherbaum, 2020; Senftleben et al., 2019). While the neural origins of value-based choice repetition effects have been rarely studied so far in humans, there is evidence that monkeys show choice repetition effects when choosing between different types of juice and that this is associated with neuronal activity in the orbitofrontal cortex (Padoa-Schioppa, 2013; Padoa-Schioppa & Assad, 2006, 2008; Rustichini & Padoa-Schioppa, 2015). Furthermore, tDCS over the mPFC in humans has been shown to alter value-based decision making through changing activity in the pretrial interval, as predicted by the same mechanisms of hyperpolarization and depolarization suggested by Bonaiuto et al. (2016) (Hämmerer et al., 2016). Hence, this leads us to assume that domain-specific neural network dynamics are involved in choice repetition processes, i.e., mPFC for *value-based* choice repetition, dlPFC for *perceptual* choice repetition. In their study, Hämmerer et al. (2016) used a risky choice task where participants chose between two options with different reward values and probabilities. The probabilities of winning the rewards changed over time. The authors measured how much participants’ choices were guided by the expected value of the options compared with how much they were guided by noise. As predicted by the same model (Bonaiuto et al., 2016), Hämmerer et al. (2016) found that depolarizing stimulation increased the influence of background noise and therefore led to an decrease in choice accuracy (i.e., participants were more random in their decision making). However, in contrast to Bonaiuto et al. (2016), the authors did not explicitly consider the influence of the previous decision and did not investigate choice repetition effects, even though this is directly predicted by the model.

Here, we close this gap and apply tDCS over the mPFC in order to alter choice repetition in *value-based* decisions. We use our previously introduced value-based decision game in which we reliably found choice repetition effects (Scherbaum et al., 2013, 2016, 2018; Senftleben et al., 2019). In that game, people have to collect coins in a two-dimensional virtual world. In each trial, they are faced with the choice between one coin that has a smaller value but is closer (i.e., quicker to collect) and another coin that has a larger value but is further away (i.e., takes longer to collect). Because people have a limited amount of time to play the game, they have to decide in each trial which coin has a higher subjective value to them (i.e., Is it worth the extra time that they would need to collect the coin with the higher value?). We now measure how making a choice in one trial is affected by the directly preceding choice. We expect that participants will show a choice repetition bias in their choices and decision times and that this choice repetition bias is increased under anodal tDCS and decreased under cathodal tDCS of the mPFC. Furthermore, we expect that decision times will be faster under anodal tDCS and slower under cathodal tDCS.

## Methods

### Preregistered hypotheses

We preregistered this study, including hypotheses and statistical analyses, at https://osf.io/rj4t7. Specifically, we expect the following hypotheses:

- H1.1) Participants show choice repetition when they do not receive any brain stimulation (sham), that is, they are more likely to repeat their previous decision than to switch to a different decision.
- H1.2) Because participants have the tendency to repeat choices, repeating the same choice leads to shorter decision times than switching to a different choice.^1^
- H2.1.1) Anodal tDCS-stimulation of the mPFC will increase the influence of the previous choice on the current decision, leading to a higher likelihood of repeating the same choice (compared with cathodal and sham stimulation).
- H2.1.2) Cathodal tDCS-stimulation of mPFC will decrease the influence of the previous choice on the current decision, leading to a smaller likelihood of repeating the same choice (compared with anodal and sham stimulation).
- H2.2.1) Anodal tDCS-stimulation of mPFC will decrease decision times (compared with cathodal and sham stimulation).
- H2.2.2) Cathodal tDCS-stimulation of mPFC will increase decision times (compared with anodal and sham stimulation).

Furthermore, we report how we determined our sample size, all data exclusions (if any), all manipulations, and all measures in the study. All data and analysis scripts are openly accessible at https://osf.io/aq9xd/.

### Participants

We expected the tDCS stimulation to have a small to medium effect size, based on effect sizes from similar tDCS-studies (Andrews et al., 2011; Casula et al., 2017; Fregni et al., 2005; Hämmerer et al., 2016). Based on power analysis using G*Power (Faul et al., 2007), we needed a sample size of 52 to detect an effect size of *d* = 0.4 with a power of 80%. We recruited participants from the ORSEE-based database (Greiner, 2015) of the Department of Psychology of the TU Dresden, Dresden, Germany (80 participants recruited; 24 participants were excluded after the measurement block due to decision making that did not allow for our experimental manipulation (see Task design); 1 participant was excluded because they were familiar with the task and the experimental design; and 3 participants were excluded due to technical difficulties with the tDCS device). The final sample consisted of 52 participants (31 females, mean age = 24.52 years, *SD* = 5.62 years). All participants had normal or corrected-to-normal vision and did not fulfill any exclusion criteria for tDCS (see supplementary materials for screening material). Participants gave informed consent and received reimbursement of 8€ per hour as well as the money that they collected within the decision task. The study was approved by the Institutional Review Board at the Technische Universität Dresden (EK 155042017) and was performed in accordance with the Declaration of Helsinki.

### Apparatus and stimuli

**Fig. 2.**
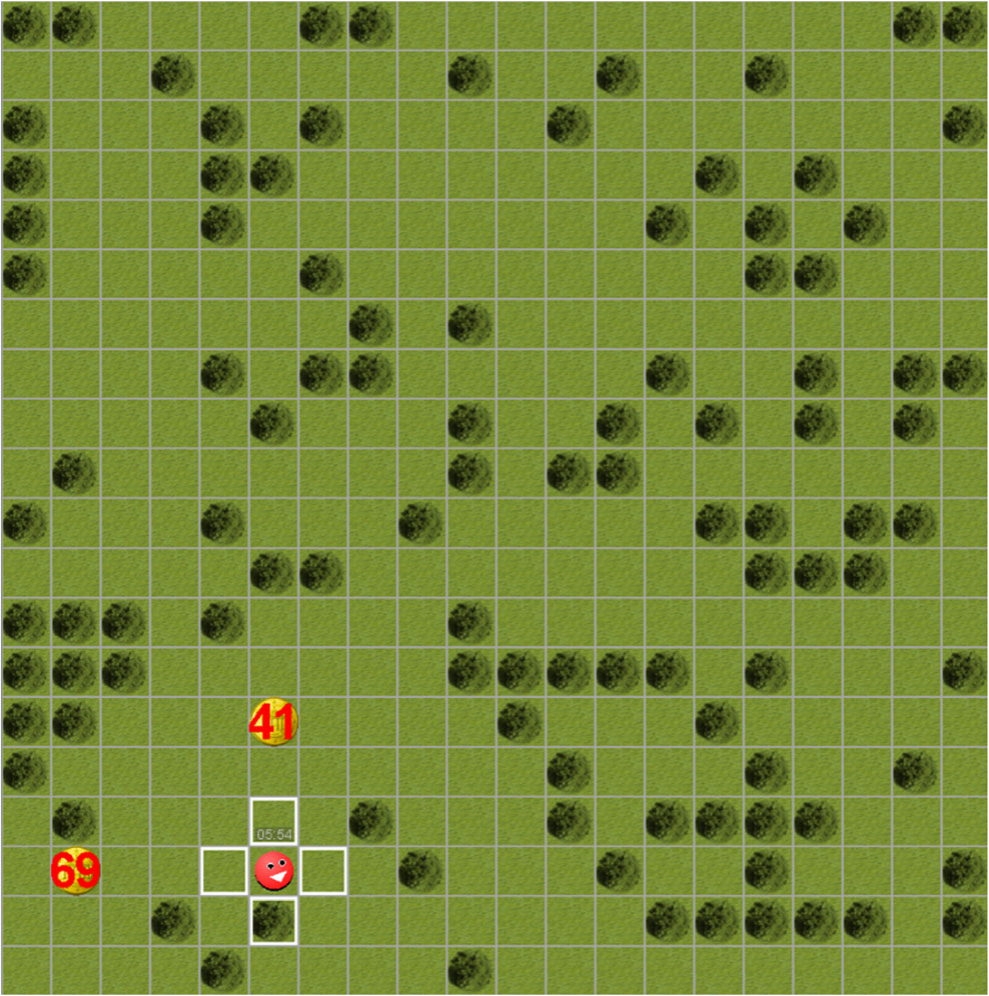
Value-based decision game. Participants controlled the red avatar by clicking into vertical or horizontal fields (outlined by white) using the computer mouse. Throughout each block, the remaining time was displayed above the avatar. Upon reaching one of the coins, both coins disappeared, and the collected credit was added to the overall accumulated credit and displayed below the avatar. The avatar remained in the position of the collected coin and could not be moved until the next trial started (i.e., two new coins appeared). Dark green fields (representing trees) were included for more intuitive spatial orientation and were not relevant to the task (i.e., they did not restrict movement and participants could freely cross these fields).

Figure 2 shows an exemplary view of the value-based decision game. The game was presented on a 17-inch screen (1280 x 1,024 pixels, 72 Hz). It was controlled using the Psychtoolbox version 3 (Brainard, 1997; Pelli, 1997) in Matlab 2006b (the Mathworks, Inc.) on a Windows XP SP2 personal computer. Participants used a Logitech USB computer mouse to make their responses. The decision game consisted of a two-dimensional world of 20 x 20 fields, with one field consisting of 50 x 50 pixels. The stimuli used within this paradigm were circular shapes with a diameter matching that of a single field (i.e., 50 pixels). Participants controlled an avatar that they could move freely from field to field by clicking with the computer mouse into either vertical or horizontal adjacent fields outlined in white. The coins used as reward stimuli were gold with the value written inside the coin in red. Throughout the whole task, the remaining time within each block was displayed above the avatar.

Two questionnaires were used in this study: the German version of the Trail Making Test (Rodewald et al., 2012) and the German version of the Action Control Scale (HAKEMP 90; Kuhl, 1994). The purpose of the questionnaires was to provide a distraction in between blocks of the experiment, they were not part of the research question.

### Procedure

**Fig. 3.**
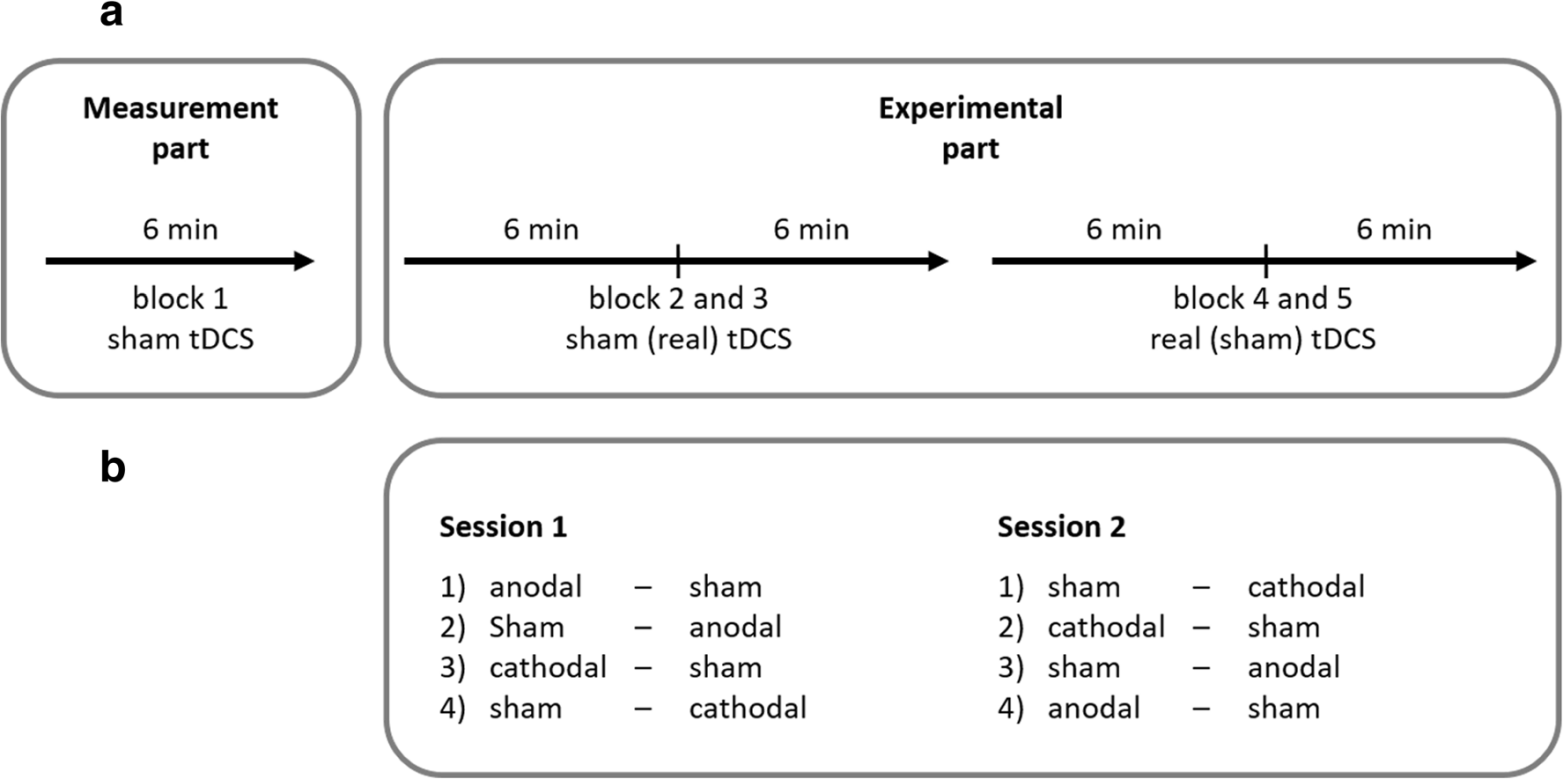
Experimental procedure. A*.* Illustration of the within-session procedure. Each session had a measurement (1 block) and an experimental part (4 blocks). Transcranial direct current stimulation (tDCS) was applied for two blocks during the experimental part (real tDCS). If real stimulation was applied during blocks 2 and 3, sham tDCS was applied during blocks 4 and 5 and vice versa. In between blocks 3 and 4, participants filled out questionnaires to provide a temporal buffer. B*.* Order of tDCS conditions within and across sessions. Each participant went through one of the four scenarios, which we balanced between participants

Participants performed the decision task while receiving tDCS stimulation in two repeated sessions. The second session took place exactly one week after the first session (same day and time). An illustration of the procedure is presented in Fig. 3. In each session, sham stimulation was started while participants read the instructions for the game and went through a tutorial (two minutes) to familiarize themselves with the task. Then they played one block of the decision game for six minutes (the so-called measurement block, see Design). We applied sham stimulation to ensure similar conditions (i.e., presence of tDCS) as later blocks of the experiment (out of concern that even sham tDCS might alter decision making).

After that, participants played another four blocks of the decision game under tDCS stimulation: two blocks under sham stimulation and two blocks under real stimulation (either anodal or cathodal). Each block lasted for 6 minutes. Participants filled in questionnaires before the first block of each stimulation (sham or real) in order to allow potential after-effects of the previous stimulation to subside. Within one session, the decision game consisted of five blocks that took 30 minutes in total (1 measurement block with sham tDCS stimulation, 4 experimental blocks with tDCS stimulation/sham). Participants could determine the length of the break in between two blocks themselves.

Within a trial of the decision game, two coins always appeared at the same time. Participants had to choose between these two coins by using the computer mouse to move the avatar field-by-field until it reached the chosen coin. Upon reaching that coin, both coins disappeared and the accumulated credit collected so far appeared. The avatar remained in the position of the last coin. The next trial started with the appearance of two new coins. The inter-trial interval (ITI) was either 500 or 1,500 ms (see Design). During the ITI, the mouse cursor was locked to the position of the avatar; it could only be moved again when the next trial started and new coins appeared.

### Task design

Our study had a within-subject design with two sessions. Participants received three different types of tDCS stimulation: cathodal mPFC stimulation, anodal mPFC stimulation, or sham mPFC stimulation. In each session, participants received sham stimulation and real stimulation (either anodal or cathodal); participants (but not experimenters) were unaware of which stimulation they received. We balanced the order of the stimulation within each session (i.e., if a participant had received the sham stimulation first in the first session, they received the real stimulation first in the second session) as well as between participants (i.e., half received anodal stimulation in the first and cathodal stimulation in the second session and half of the participants vice versa).

In each session, participants played the decision game. The game was split into two different parts: a measurement part intended to measure participants’ individual decision preferences (one block of 6 minutes), and an experimental part intended to induce choice repetition (four blocks of 6 minutes). For both parts, trials were constructed so that one coin was closer to the avatar but had a smaller value (small/near (SN) option), and the other coin was further away but had a larger value (large/far (LF) option). Coins were situated at least 90 degrees away from each other with respect to the avatar. This ensured that moving toward one coin increased the distance to the other one. One credit in the game equaled 0.01 € in real money, which participants received after the experiment.

The measurement part of the task consisted of one block of trials with a wide range of combinations of distances and values. The SN option was either 2 or 3 fields away from the avatar, the LF option was either 1, 4, 8, or 12 fields further away from the avatar than the SN option (this variable is called *distance* from here on). In each trial, the value of the LF option was drawn randomly from a range of 65 to 85 credits, whereas the value of the SN option randomly varied between 20% to 95% of the LF option. During the measurement block, the ITI was kept constant at 500 ms.

We used the choice data of this measurement block to estimate participants’ indifference points. Indifference points describe the specific ratio of SN to LF value where both options are equally attractive to the participant (i.e., the probability of choosing either the LF or SN option is 50% respectively). For each participant, we calculated indifference points for each distance (1, 4, 8, 12 fields) (for details, see the Supplement). Based on these four indifference points for the four distances used in the measurement block, we interpolated the indifference points for the remaining distances between 1 and 12.^2^ As a result, we obtained estimates of indifference points for each distance between 1 and 12, which we then used to construct trials in the subsequent experimental part. If participants discounted very highly or not all—specifically, if participants’ discounting did not allow to us to create trials with values 30% above or below the indifference points for at least two different distances—the experiment ended after the measurement block because the manipulation of the experimental blocks would not have been possible.

The experimental part consisted of four blocks. We built triplets of trials where the first two trials served as bias trials (SN or LF) and the third trial as a neutral target trial. For the bias trials, we chose reward values so that either the SN option or the LF option was more attractive. We achieved this by setting the value of the SN option either to 30% above the indifference point, making the SN option more attractive, or by setting the value of the SN option to 30% below the indifference point, making the LF option more attractive. Based on participants’ discounting, we identified for which distances this manipulation was possible (as the indifference point had to be either below 0.7 for attractive SN trials or above 0.3 for attractive LF trials) and drew randomly from these distances for each bias trial. The value of the LF option ranged from 65 to 85.

We constructed neutral target trials—that is, trials where participants were equally likely to choose either option—by setting the value of the SN option to the indifference point. Distances for neutral target trials were drawn randomly from a range of 1 to 12 fields.

We also varied the ITI between trials. We used a short ITI (500 ms) and a long ITI (1,500 ms). The ITI between the second bias trial and the target trial was always short to maximize the effect of the bias since choice repetition is strongest for short ITIs (Bonaiuto et al., 2016; Senftleben et al., 2019). For the other two ITIs (target trial to first bias trial; first bias trial to second bias trial), one ITI was short and one was long, but the order was randomized in order to mask the triplet-structure of the trials from participants.

### Transcranial direct current stimulation

**Fig. 4.**
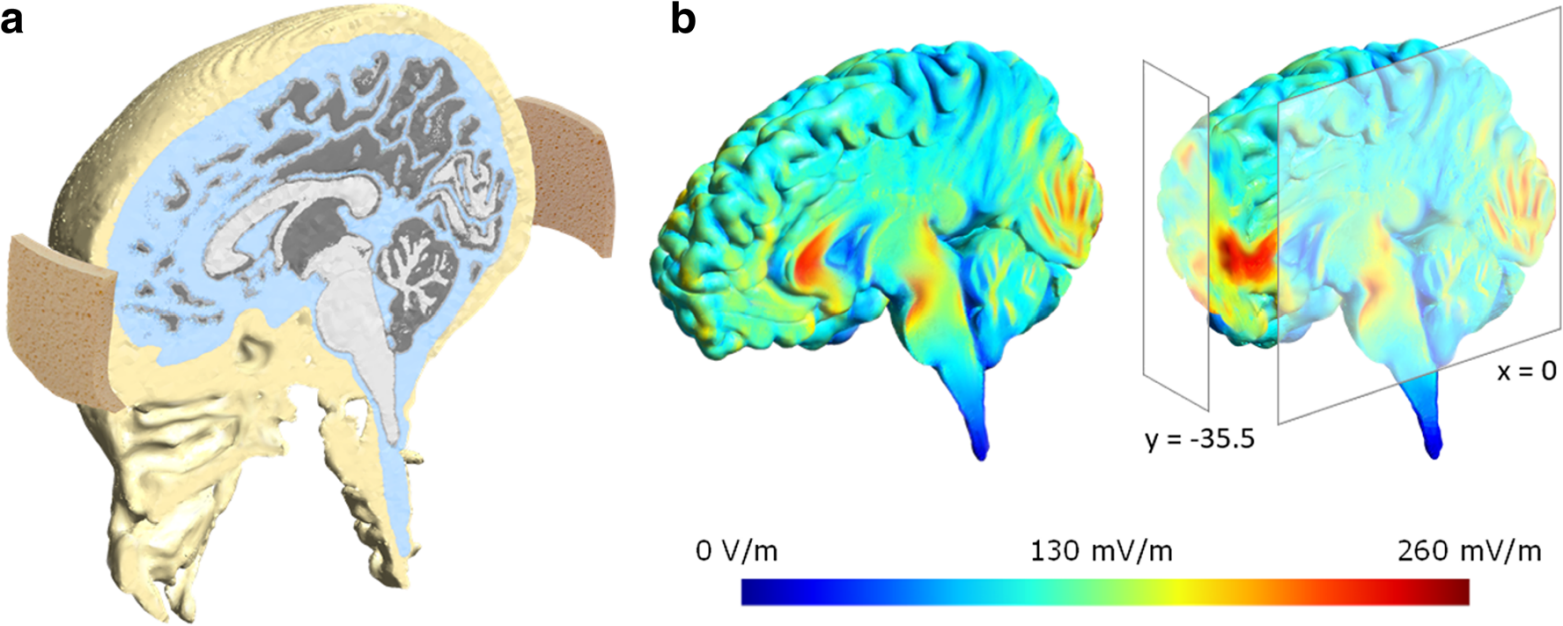
tDCS current simulation. A. Illustration of the electrode placement. 5 x 5 cm electrodes were placed over Fpz and just below the inion, roughly corresponding to Oz. B. Simulated current flow at 1 mA showing maximum current at targeted region, i.e., mPFC at sagittal midline view (left panel) and frontal brain view (right panel; cut-out at x = 0 and y = −35.5)

We used a battery driven constant current stimulator (DC-Stimulator Plus, neuroConn GmbH, Germany) and two conductive 5- x 5-cm rubber electrodes. The electrodes were inserted in synthetic sponges that were soaked in saline solution and held in position by a headband. We had three different stimulation conditions: anodal mPFC stimulation, cathodal mPFC stimulation, and sham mPFC stimulation. We placed the electrodes in the same montage as reported by Hämmerer et al. (2016) and verified mPFC targeting. To this end, we simulated current flow using SimNIBS (SimNIBS 3.1.2 and gmsh 3.0; Saturnino et al., 2019), GetFEM (Renard & Poulios, 2020), and Python code (for results, see Fig. 4). For anodal mPFC stimulation, the anodal electrode was placed over electrode Fpz according to the international EEG 10-20 system while the reference electrode was placed just below the inion, roughly corresponding to Oz. For cathodal mPFC stimulation, electrode placement was reversed. For sham stimulation, we used the same electrode placement as in anodal mPFC stimulation.

For each active stimulation condition, stimulation was applied at a constant current of 1 mA for 12 minutes (equals a current density of 0.04 mA/cm^2^), with additional fade in and fade out times of 30 seconds. Stimulation started 2 minutes before the task. For sham stimulation, stimulation was applied at a constant current of 1 mA for 30 seconds, with fade in and fade out times of 30 seconds. This was to ensure that participants felt the tingling sensation that can occur in the first seconds of tDCS stimulation, so that they were kept unaware of the sham condition (Gandiga et al., 2006).

### Data analysis

Data processing was carried out in Matlab 2017b running on a Windows 10 computer. Further statistical analysis was carried out in JASP version 0.11.1 (JASP Team, 2019). Decision times were log-transformed for statistical analysis in order to reduce skewness.

## Results

### Behavioral results

Results of the measurement block are summarized in the Supplement. We performed a manipulation check which showed that we successfully manipulated the subjective values according to the trial type (i.e., participants chose the LF option in LF trials, the SN option in SN trials, and were indifferent in neutral target trials), which is presented in Figure S2 in the Supplement. In the experimental block, participants completed an average of 479.55 trials (*SD* = 77.87 trials) in session 1 and 492.71 trials (*SD* = 86.67 trials) in session 2.

**Fig. 5.**
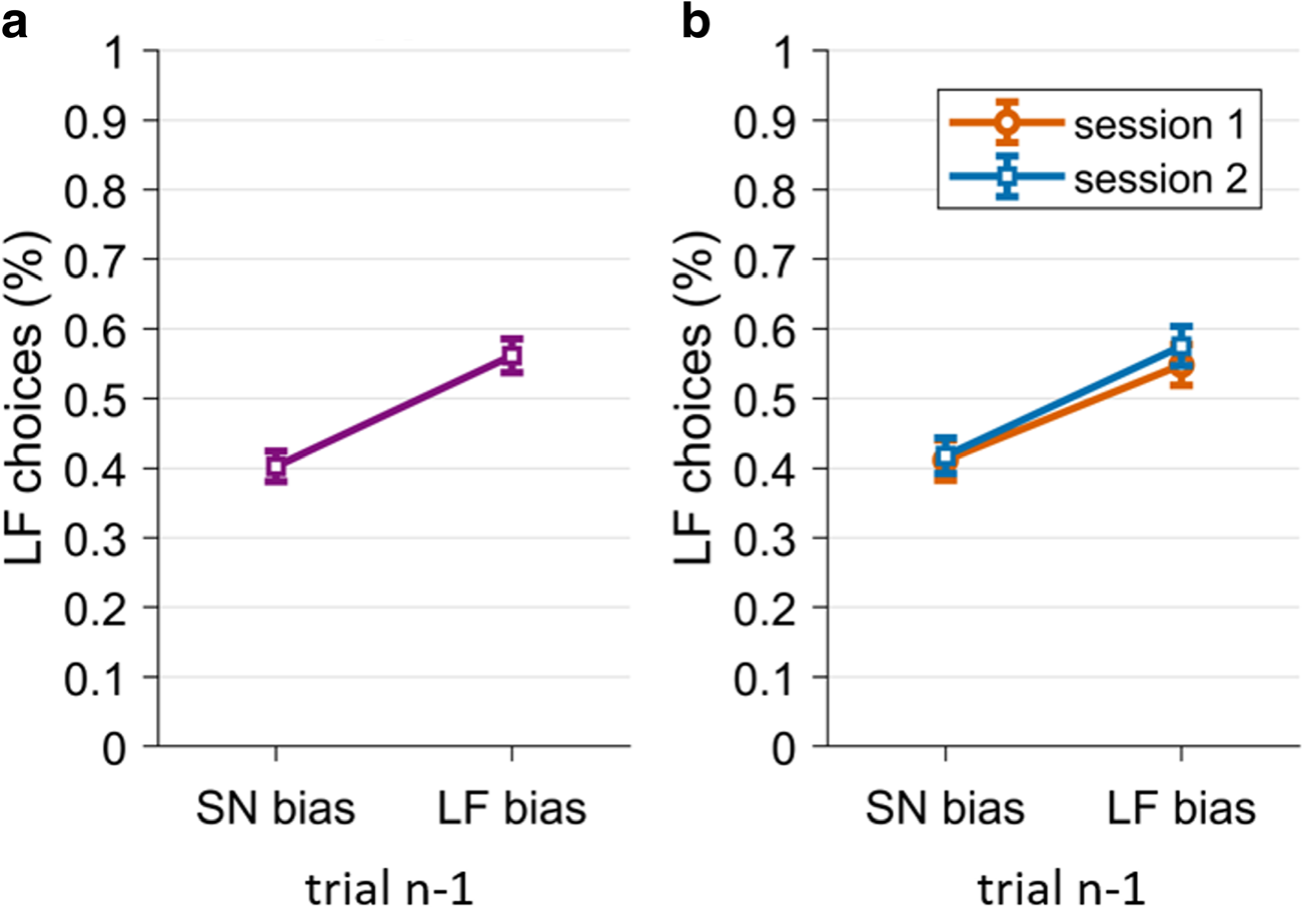
Choice repetition effect. Percentage of large/far (LF) choices under sham stimulation, after small/near (SN) or LF bias choice. Panel A depicts this effect averaged across both sessions, Panel B depicts this effect separately for session 1 (orange circles) and session 2 (blue squares). Error bars represent standard errors

To test for choice repetition in each session, we calculated the percentage of LF choices in target trials separately for SN bias or LF bias in the preceding bias trial. We only included trials from the sham stimulation blocks and trials where the bias manipulation was successful (i.e., SN choice in SN bias trial, LF choice in LF bias trial; mean success rates of the bias manipulation were 80.21% of trials in session 1 (*SD* = 7.66%) and 79.71% of trials in session 2 (*SD* = 7.70%)). As planned in the preregistration, we pooled trials across sham sessions because there were no significant differences between LF choice percentages between conditions, *t*(51) = −1.13, *p* = 0.27, *g* = −0.17. We expected choice repetition to lead to more LF choices in target trials after an LF bias trial compared with target trials after an SN bias trial (H1.1). Indeed, the percentage of LF choices was significantly higher after LF bias trials (*M* = 56.15%, *SD* = 17.53%) compared with after SN bias trials (*M* = 40.24%, *SD* = 15.51%), *t*(51) = 7.76, *p* < 0.001 (one-tailed), *g* = 0.95; see Fig. 5A. Exploratory analyses showed that this choice repetition effect was present in both sessions (Fig. 5B) and in the vast majority of participants (79% in the first session, 83% in the second session; see Figure S3 in the Supplement). The repetition index was correlated across sessions (*r* = 0.28, *p* = 0.04), hinting to some degree of within-subjects stability.^3^ Hence, participants showed choice repetition as expected and this effect was robust in both sessions.

**Fig. 6.**
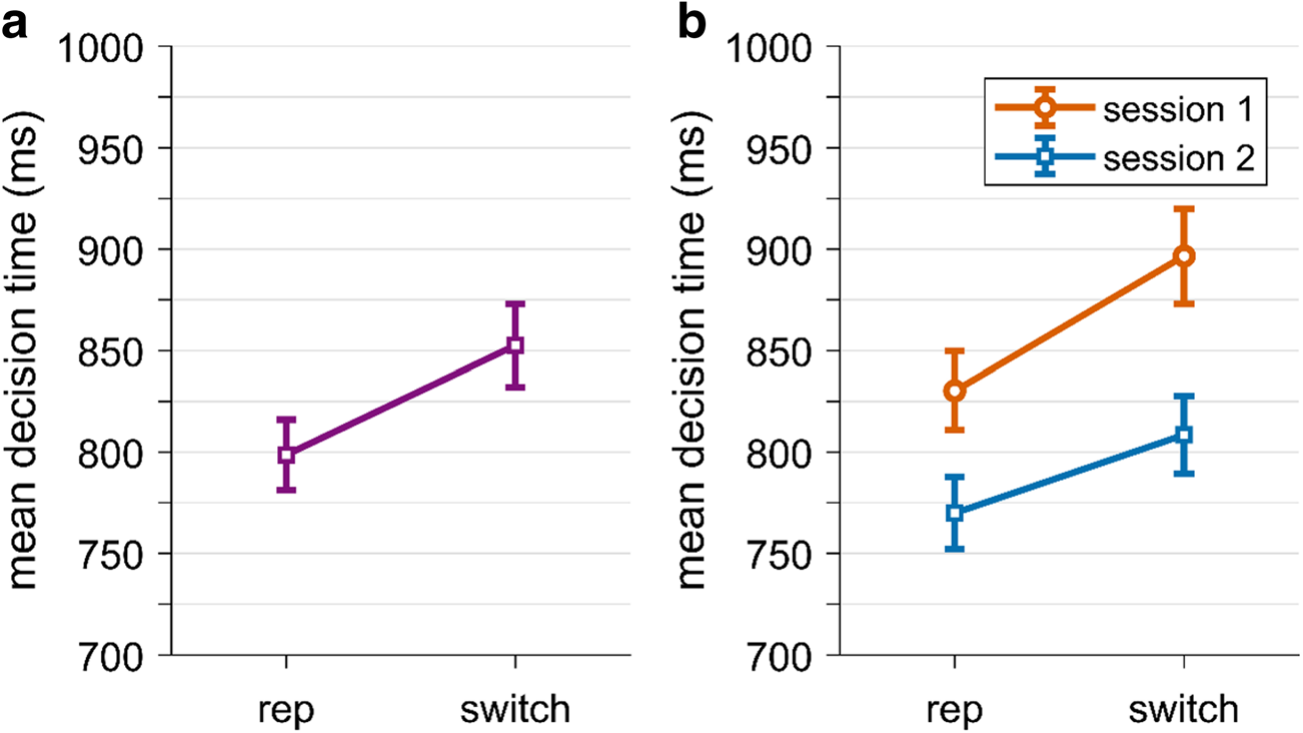
Decision times for target trials under sham stimulation, for repetitions (rep) of the bias choice and for switches from the bias choice. A. Effect averaged across both sessions, while B) shows this effect separately for session 1 (orange circles) and session 2 (blue squares). Error bars represent standard errors

Next, we tested if choice repetitions were faster than choice switches (H1.2). We analyzed log-transformed decision times in target trials where participants either repeated the choice from the bias trial or where they switched to the other choice option. In parallel to the choice repetition analysis described above, we only included trials from sham stimulation and trials where the bias trial manipulation was successful. Averaged across sessions, we found that repetitions (*M* = 798.65 ms, *SD* = 125.12 ms) were indeed significantly faster than switches (*M* = 852.61 ms, *SD* = 147.37 ms), *t*(51) = 9.09, *p* < 0.001, *g* = 0.39; see Fig. 6A. Hence, we found support for our hypothesis that repetitions are easier and therefore faster than switches. Interestingly, we also found that decision times differed across sessions (Fig. 6B). While we had no prior hypotheses on session effects regarding decision times, we ran a 2x2 factorial repeated measures ANOVA with the factors Session (1 or 2) and Type (repetition or switch) to test this.^4^ (For descriptive statistics, see Table S2 in the Supplementary Materials). The analysis revealed a significant main effect of Type, *F*(1,51) = 87.87, *p* < 0.001, η_p_^2^ = 0.63, which is in line with our hypothesis of faster decision times for repetitions. Post hoc *t*-tests confirmed that this effect was significant in each session, *p*s < 0.001 (Holm-corrected). The analysis also revealed a significant main effect of Session, *F*(1,51) = 35.27, *p* < 0.001, η_p_^2^ = 0.41, indicating that participants were faster in the second session compared with the first session. The interaction Type x Session was close to significance, *F*(1,51) = 3.65, *p* = 0.06, η_p_^2^ = 0.07, which might indicate that the decision time effect of repetitions vs. switches was attenuated in the second session.

In summary, we found support for both our choice repetition hypotheses: Participants tended to repeat their choices, and they were faster when repeating choices. We also conducted exploratory analyses, which showed that decision times were generally faster in the second session (possibly indicating familiarity with the paradigm/training effects), and that the decision time advantage of repetitions over switches was somewhat attenuated in the second session.

### tDCS results—confirmatory analyses

**Fig. 7.**
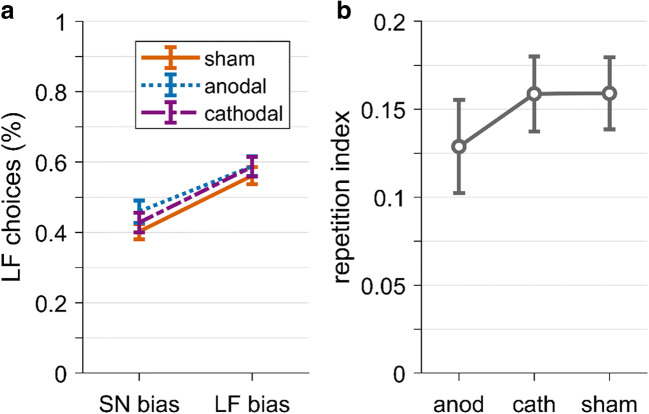
Effect of tDCS stimulation on choice repetition. A. Percentages of large/far (LF) choices in target trials, after small/near (SN) or LF bias choice. Cathodal = purple dashed line, anodal = blue dotted line, sham = orange solid line. B*.* Perseveration index for each tDCS stimulation. The repetition index is the difference of LF choice percentage after LF bias and LF choice percentage after SN bias. Anod = anodal, cath = cathodal. Error bars represent standard errors

We expected tDCS stimulation to affect choice repetition (hypothesis 2.1). Specifically, we expected choice repetition to increase under anodal stimulation and to decrease under cathodal stimulation compared with sham (hypotheses 2.1.1 and 2.1.2). Because participants underwent the sham condition in both sessions and there were no significant differences in choice behavior between them (see Behavioral results), we pooled data from both sessions for the sham condition. To test our hypothesis that tDCS alters choice repetition, we first calculated a repetition index. For this repetition index, we took the percentage of LF choices in target trials separately for trials following SN bias choices and for trials following LF bias choices; we only included trials with a successful bias manipulation (see Behavioral results). We then calculated the difference between these two LF percentages for each participant; the bigger this difference is, the stronger is the choice repetition. We calculated this repetition index separately for each tDCS condition (Fig. 7). We then ran a one-way repeated measure ANOVA with the repetition index as outcome and with the factor tDCS (anodal, cathodal, sham). Contrary to our hypothesis, the main effect of tDCS was not significant, *F*(2,102) = 1.00, *p* = 0.37, η^2^ = 0.007. Post hoc t-tests of anodal vs. sham and cathodal vs. sham were also not significant, *p*s > 0.56. Finally, we ran a Bayesian repeated measures ANOVA that revealed evidence in favor of the null hypothesis (BF_01_ = 6.73; for details, see Supplementary Materials Table S3). Hence, we did not find any effect of tDCS on choice repetition and could not confirm hypothesis 2.1.

**Fig. 8.**
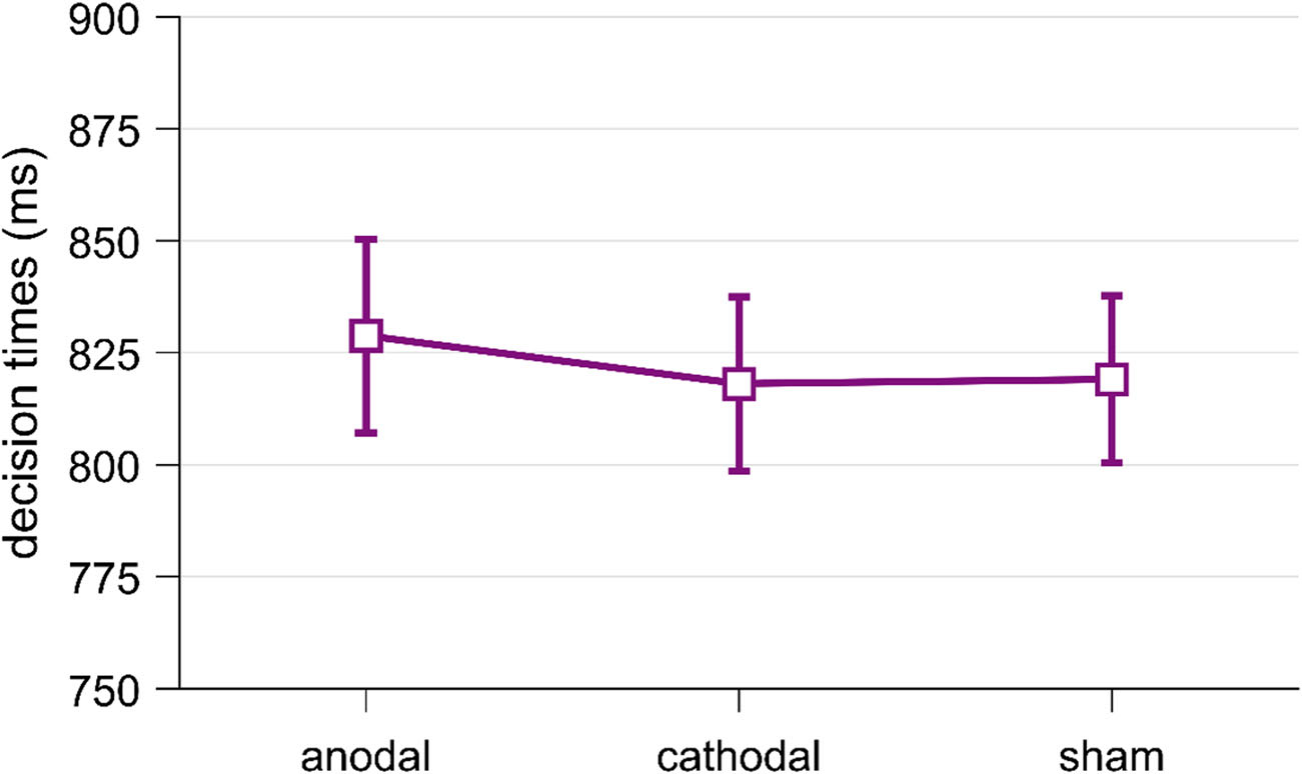
Decision times in milliseconds across all target trials, separately for anodal, cathodal, and sham stimulation. Error bars represent standard errors

Our second hypothesis about tDCS was that tDCS alters decision times (hypothesis 2.2). Specifically, we predicted that anodal tDCS decreases decision times (H2.2.1) and that cathodal tDCS increases decision times (H2.2.2). We again pooled data from the sham conditions of both sessions for each participant. We log-transformed decision times to reduce skewness, and we included decision times from all target trials. We analyzed the log-transformed decision time by running a repeated measures ANOVA with the factor tDCS (anodal/cathodal/sham). Against our expectations, tDCS had no significant effect on decision times, *F*(2,102) = 0.31, *p* = 0.73, η^2^ = 0.01; see Fig. 8. Post hoc *t*-tests of anodal vs. sham and cathodal vs. sham were also not significant, *p*s >0.41. We further ran a Bayesian repeated measures ANOVA that revealed evidence in favor of the null hypothesis (BF_01_ = 12.15; for details, see Supplementary Materials Table S4).

### tDCS results – exploratory analyses

**Fig. 9.**
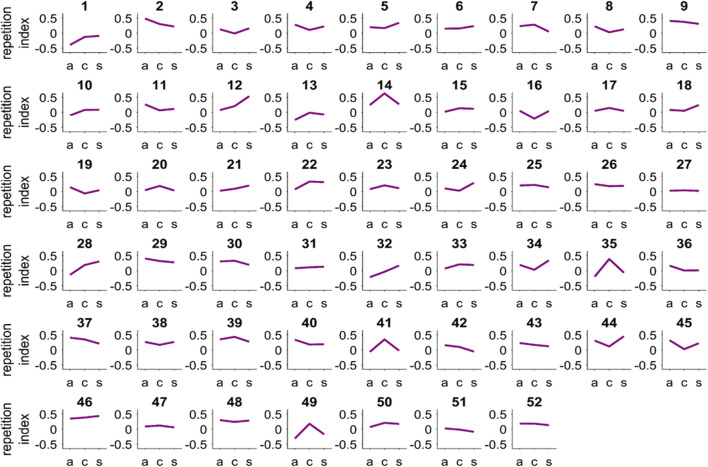
Repetition index for each tDCS condition (a: anodal; c: cathodal; s: sham), displayed for each of the 52 participants. The repetition index is defined as the percentage of LF choices after LF bias trials minus the percentage of LF choices after SN bias trials (a positive value indicates choice repetition)

We ran additional analyses to further investigate our tDCS null effects. First, we looked at the single-subject level in order to understand the nature of the tDCS effect on choice repetition. We thought it possible that perhaps tDCS modulated choice repetition in some but not in all participants. However, when looking at the effect of tDCS on the repetition index in all 52 participants, we did not find any evidence indicating a systematic influence of tDCS; rather, there was a considerable amount of interindividual variance that did not reveal a homogeneous pattern (Fig. 9).

**Fig. 10.**
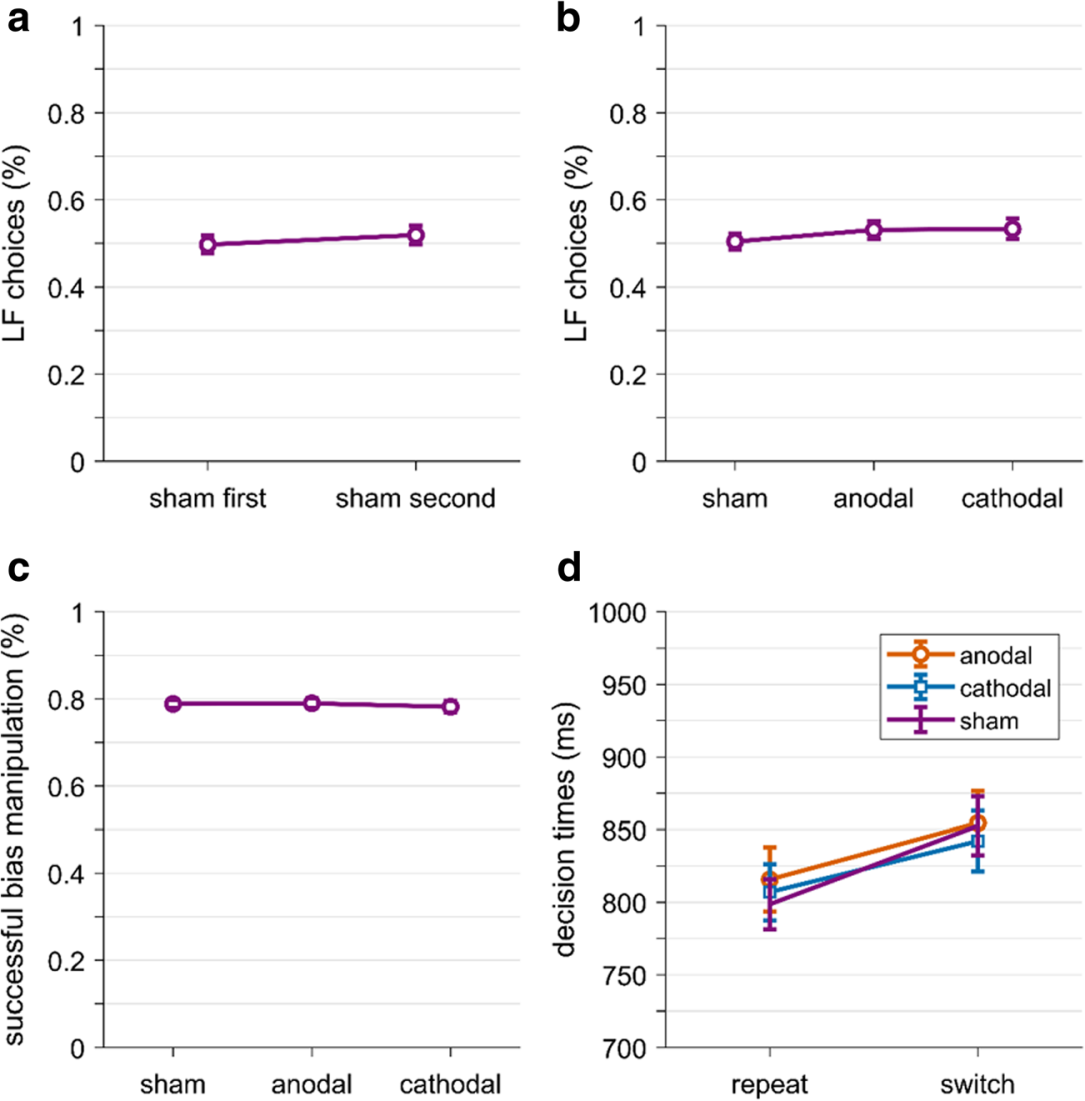
Results of exploratory analysis of tDCS. **A**) Choice percentages for the large/far (LF) option across all trials in the experimental blocks for each sham sessions. Sham first means that participants first received sham, then real stimulation; sham second means that participants first received real, then sham stimulation. **B**. LF choice percentages across all trials in the experimental block for each tDCS condition. Trials in the sham condition were pooled over the sham blocks of both sessions. **C**. Percentage of successful bias manipulation, that is, bias trials where participants chose the option that was more valuable (SN choice in SN bias trials, LF choice in LF bias trials), for each tDCS condition. Sham conditions were pooled. D) Mean decision times across target trials that were either a repetition of the previous trial (repeat) or a switch, for each tDCS condition (sham data pooled across sessions). Error bars represent standard errors

Next, we explored potential effects of tDCS on overall choice behavior. First, we wanted to make sure that our pooled sham condition was appropriate and not affected by potential after-effects of tDCS. Although we did not find any difference between the two sham sessions for target trials, we wanted to look at choices in all trials of the experimental block. Hence, we compared overall LF choice percentages between the sham session where sham stimulation was first (before real stimulation) with the sham session where the real stimulation came first. We found no difference between the two conditions, *t*(51) = 0.98, *p* = 0.33, *g* = 0.14 (Fig. 10A). Hence, there is no evidence that the sham condition that occurred after real stimulation was influenced by after-effects of tDCS. Second, we checked whether tDCS affected overall choice preferences, regardless of trial type. Therefore, we ran a repeated measures ANOVA with the factor tDCS (sham, anodal, cathodal) on overall LF choice percentage across all trials in the experimental blocks (bias and target trials). Again, we found no evidence for an effect of tDCS, *F*(2,102) = 1.49, *p* = 0.23, η^2^ = 0.008 (Fig. 10B). Third, we explored whether tDCS had an effect on our trial type manipulation, that is, whether tDCS altered participants’ sensitivity to the subjective value manipulation (Fig. 10C). We calculated the biasing success rate (i.e., how often did participants choose the SN option in SN bias trials and the LF option in LF bias trials) and ran a repeated measures ANOVA with the factor tDCS (sham, anodal, cathodal). There was no significant effect of tDCS, *F*(2,102) = 0.25, *p* = 0.78, η^2^ = 0.002.

Finally, we looked at decision times (Fig. 10D). While our confirmatory analysis did not reveal an impact of tDCS on overall decision times, we were interested in looking at the effect of tDCS on the switch costs we found between choice repetitions and choice switches. We ran a repeated measures ANOVA with the factors tDCS (sham, anodal, cathodal) and trial type (switch, repetition) on decision times in target trials. Neither the main effect of tDCS, *F*(2,102) = 0.37, *p* = 0.70, η^2^ = 0.001, nor the interaction, *F*(2,102) = 1.33, *p* = 0.27, η^2^ = 0.001, was significant. The main effect for trial type was significant as expected, *F*(2,102) = 57.05, *p* < 0.001, η^2^ = 0.02. Overall, our exploratory analyses did not reveal any further indications of tDCS effects on behavior.

## Discussion

In this study, we investigated whether choice repetition effects in value-based choices can be altered through transcranial current stimulation (tDCS) over the medial prefrontal cortex (mPFC). While we found model-predicted choice repetition effects both on choice and on response times, we did not find evidence for an effect of tDCS.

### Choice repetition bias in value-based decisions

Our study shows that, as expected, people have a tendency to repeat their choices in a value-based decision task. This finding is in line with our previous studies using the same decision task, where we gradually made one choice option less and less valuable and measured how long participants would stick to that choice option (due to choice repetition tendencies) (Scherbaum et al., 2016; Schoemann & Scherbaum, 2020; Senftleben et al., 2019). Our present study shows that choice repetition is not merely a by-product of longer, gradual decision sequences; rather, choice repetition also occurs on a trial-by-trial basis. This effect was equally strong in both sessions, indicating its robustness. Furthermore, acting against this choice repetition bias comes at a cost, as indicated by slower decision times when not repeating the previous choice. This decision time effect seems more prone to practice effects, as it was attenuated (but still present) in the second session. In the context of our paradigm, where participants have to collect as much rewards as possible under time pressure, longer decision times have direct costs for the overall task performance and task rewards. Hence, this might indicate that repeating decisions might be an advantageous strategy (as it minimizes decision time), even though simply repeating decisions can lead to choosing the less valuable option.

Our results are in line with other empirical and modeling work on decision sequence effects in both value-based (Alós-Ferrer et al., 2016; Hämmerer et al., 2016; Rustichini & Padoa-Schioppa, 2015) and perceptual decision making (Berlemont & Nadal, 2019; Bonaiuto et al., 2016; Braun et al., 2018; Gao et al., 2009; Nguyen et al., 2019; Urai et al., 2019). Hence, while this phenomenon has primarily received attention in the field of perceptual decision making, our present study and a growing body of research highlights that decision sequence effects, such as choice repetition, play a role in various domains of decision making. Therefore, research on economic and value-based decision making should take this into account when modeling and predicting choice behavior, instead of merely focusing on isolated decisions.

### No influence of tDCS on choice repetition

Against our expectations, we did not find any effect of medial frontal tDCS on choice repetition. This goes against previous results in a perceptual decision-making task (Bonaiuto et al., 2016). Those authors applied tDCS over the left dorsolateral prefrontal cortex (dlPFC) while participants performed a random dot kinematogram task (identifying if there is coherent movement to the left or right in a group of mostly randomly moving dots). The authors found choice repetition effects, such that participants were more likely to perceive movement to the left if there was movement to the left in the previous trial. Crucially, tDCS affected this choice repetition. As predicted by a computational neural network model, anodal tDCS (which is thought to depolarize the network) increased the influence of the previous decision, whereas cathodal tDCS (which is thought to hyperpolarize the network) decreased the influence of the previous decision. Furthermore, Hämmerer et al. (2016) demonstrated similar effects in a value-based decision task while applying tDCS over the mPFC. In their experiment, they presented participants with a risky choice task where the probabilities for each option changed over time. They measured how much participants’ decision making was driven by the expected value of the options versus by random noise. While the authors did not specifically investigate choice repetition effects, they showed that anodal tDCS over mPFC (but not sham or dlPFC stimulation) affected choice accuracy by amplifying the influence of background noise in the neural system, particularly in the time period before the current trial. While inter-trial effects were not investigated, this would be consistent with an increased influence of the previous decision (as the decaying activity from the previous trial affects activity levels in the time period before the current trial).

Therefore, it is surprising that we did not find any influence of tDCS on choice repetition, which leads us to speculate about potential explanations for this null effect. We cannot say for certain that choice repetition effects, such as studied here, originate in the mPFC, because this has not been studied before in humans. Perhaps we did not find any effects of mPFC stimulation on choice repetition, because we simply did not stimulate the causally responsible brain region. While the mPFC is responsible for value calculation and therefore choice, it may be that value-based choice repetition originates in the dlPFC instead (as Bonaiuto et al. (2016) showed for perceptual decisions). In other words, perhaps choice repetition is a domain-general process that is located in an executive control area, such as the dlPFC, and not a domain-specific process that is located in the respective processing area (as we assumed). However, there are two arguments to be made against this point. First, choice repetition effects naturally emerge from neural network dynamics caused by excitation and inhibition. These network dynamics have been demonstrated to occur in the mPFC. Specifically, mPFC activity during value-guided choice has been linked to such neural network dynamics caused by mutual inhibition and self-excitation in a magnetoencephalography study with human participants (Hunt et al., 2012). This is corroborated by evidence showing that the levels of inhibitory and excitatory neurotransmitters (GABA and glutamate) influence value-based decision making in humans as predicted (Jocham et al., 2012). In addition, studies in nonhuman primates have clearly demonstrated choice repetition effects that are tracked by neurons in the orbitofrontal cortex (Padoa-Schioppa & Assad, 2006, 2008). Second, the null effects were not specific to choice repetition. Rather, we did not find any effect of tDCS on decision making at all. The evidence for the role of mPFC in value-guided choice is overwhelming, as reviewed in the introduction. Furthermore, medial frontal tDCS has been shown to affect behavior in various different value-based decision paradigms, such as probabilistic decision making (Casula et al., 2017), risky decision making (Hämmerer et al., 2016; León et al., 2020), and delay discounting (Manuel et al., 2019). Hence, successful mPFC stimulation realistically should have had some impact on choice preferences or decision times, but we did not find any evidence of that.

This leads us to speculate about potential methodological explanations for these null effects. To discern potential explanations, we compare our study to Hämmerer et al. (2016). The obvious differences are 1) that our design was slightly different and 2) that our tDCS stimulation was of weaker intensity. We will discuss these points as potential limitations in more detail below.

The first divergence is our research design. We implemented anodal, cathodal, and sham stimulation in a within-subjects design with two separate sessions (whereas Hämmerer and colleagues compared anodal mPFC stimulation to dlPFC stimulation and no stimulation in separate within-subjects session). In each session, participants received both sham and real (block-wise) stimulation, with the order of sham and real stimulation balanced within and between subjects. This means that for each participant, they had one session where the sham stimulation came before the real stimulation (sham-first) and one session where the real stimulation came before the real stimulation (sham-second). Because tDCS stimulation has been shown to have after-effects (Nitsche & Paulus, 2001), it is possible that participants were still influenced by the preceding real stimulation in the sham-second condition. In that case, the data collected under the sham-second condition would not constitute actual sham data. This could potentially play a role in our null findings, because we pooled sham data across both sessions. However, we did not find any differences in decision making between the sham-first and sham-second conditions. We also did not find any difference between cathodal and anodal stimulation (which took place in separate sessions and could not have been affected by any after-effects). Hence, it is implausible that this null effect is simply a by-product of our research design.

The second divergence is the tDCS protocol itself. Our electrode placement and sizes were the same as used by Hämmerer et al. (2016), which is a comparable setup to other medial frontal tDCS studies (Adenzato et al., 2017; Liao et al., 2018; Wang et al., 2020; Zheng et al., 2016). However, we used a lower current intensity of 1 mA (compared with 2 mA used by Hämmerer et al. (2016)). We chose the lower current intensity after all participants of a pilot study correctly indicated when they received real compared with sham stimulation, even with a gradual increase of current intensity in both the real and sham stimulation conditions as suggested in the literature (Gandiga et al., 2006). We wanted to ensure that participants remained blind to the tDCS conditions and that they did not experience any strong side effects that might in turn affect their behavior. Therefore, we decided to use a current intensity of 1 mA, yielding a current density of 0.04 mA/cm^2^. This is a common current density strength that has been used in various other tDCS studies (Bogdanov et al., 2015; Gandiga et al., 2006; Hummel et al., 2005; Hummel & Cohen, 2005; Iyer et al., 2005; Yuan et al., 2017), and significant effects of tDCS on cognition have even been reported for lower current densities, such as 0.029 mA/cm^2^ (for a review, see Nitsche & Paulus, 2011). However, current density and focality is a topic of ongoing research and discussion. For example, there is considerable intra- and interindividual variability in how people respond to tDCS stimulation (Chew et al., 2015; Fig. 9), and there is an ongoing debate on the specifics of electrode sizes and setups (e.g., standard vs. high-definition tDCS, cephalical or extra-cephalical reference electrodes; see Nitsche & Paulus, 2011). Furthermore, it is unclear whether the relationship between electric current density and cortical excitability is linear or nonlinear (Bastani & Jaberzadeh, 2013; Batsikadze et al., 2013; Ho et al., 2016; Kidgell et al., 2013). In addition, the effects of current density might depend on the targeted brain area. Reflecting this uncertainty, a recent guide on conducting tDCS studies recommends that stimulation duration and current density should “replicate similar protocols that have stimulated the same target region as the proposed experiment” (Thair et al., 2017, p. 5). Taking a closer look at publications reporting significant effects of medial frontal tDCS on cognitive processing, current densities of 0.057 mA/cm^2^ or higher dominate the field in recent years (Casula et al., 2017; Chib et al., 2013; Civai et al., 2014; Hämmerer et al., 2016; Liao et al., 2018; Mainz et al., 2020; Manuel et al., 2019; Zheng et al., 2016). Amongst the few studies reporting lower current densities, one did not find any effects of anodal or cathodal tDCS on performance in a trust game (Colzato et al., 2015). Two other studies reported mixed results based on gender effects and direction of current flow (Adenzato et al., 2017; Wang et al., 2020). To our knowledge, so far there is no systematic investigation of current strength density in medial frontal tDCS and its effects on decision making. However, a study on working memory performance directly compared the effect of tDCS over the dlPFC under low (0.029 mA/cm^2^) and high (0.057 mA/cm^2^) current density and found a significant effect only for high current density (Teo et al., 2011). The same pattern was found for tDCS over the right intraparietal sulcus in a visual attention task, where again only a current density of 0.057 mA/cm^2^ (compared with 0.029 mA/cm^2^) had a significant effect on performance (Moos et al., 2012). Taken together, this leads us to speculate that for medial frontal tDCS, current densities of 0.04 mA/cm^2^ may be insufficient to affect cortical excitability reliably in a majority of people; hence, interindividual variability, as we found in our study (Fig. 9), may then lead to a null effect. Future research is necessary to clarify this 1) by systematically investigating the effect of current density on medial frontal cortex excitability, and 2) by replicating the current study with a stronger current density. Attention needs to be paid to potentially stronger side-effects and their consequences for participants’ blindness to the study protocol.

## Conclusions

In our preregistered study, we show that humans display a robust choice repetition bias in value-based decision making. Specifically, this is reflected in faster decision times and in a higher frequency of choice repetitions as compared to switches. We did not find any effect of medial frontal tDCS on decision making. This may be a consequence of interindividual variability in the tDCS effect and its interaction with current strength density or may question the robustness of medial frontal brain involvement in this type of decision task.

## Supplementary Information

ESM 1 (PDF 375 kb)

## Footnotes

1 Please note that this hypothesis was not pre-registered; we decided to investigate this hypothesis before data analysis in order to replicate similar findings from previous studies (e.g., Senftleben et al., 2019)

2 We interpolated in the following ways: If the measured indifference points were monotonically decreasing, we interpolated cubically using the “pchip” method within the function interp1 in Matlab. If the measured indifference points were not monotonically decreasing, we fit them to an exponential and a hyperbolic model. The model that provided the better fit (as quantified by standard errors) was used to interpolate the indifference points.

3 Please note that this is an exploratory analysis and this study was not designed (and not powered) for correlation analyses between sessions.

4 Please note that this analysis is of exploratory nature, as we did not have any hypotheses for this session effect

## References

[CR1] Adenzato M, Brambilla M, Manenti R, De Lucia L, Trojano L, Garofalo S, Enrici I, Cotelli M (2017). Gender differences in cognitive Theory of Mind revealed by transcranial direct current stimulation on medial prefrontal cortex. Scientific Reports.

[CR2] Alós-Ferrer C, Hügelschäfer S, Li J (2016). Inertia and decision making. Frontiers in Psychology.

[CR3] Andrews SC, Hoy KE, Enticott PG, Daskalakis ZJ, Fitzgerald PB (2011). Improving working memory: The effect of combining cognitive activity and anodal transcranial direct current stimulation to the left dorsolateral prefrontal cortex. Brain Stimulation.

[CR4] Bastani A, Jaberzadeh S (2013). Differential Modulation of Corticospinal Excitability by Different Current Densities of Anodal Transcranial Direct Current Stimulation. PLoS ONE.

[CR5] Batsikadze G, Moliadze V, Paulus W, Kuo MF, Nitsche MA (2013). Partially non-linear stimulation intensity-dependent effects of direct current stimulation on motor cortex excitability in humans. Journal of Physiology.

[CR6] Berlemont K, Nadal J-P (2019). Perceptual decision making: Biases in post-error reaction times explained by attractor network dynamics. The Journal of Neuroscience.

[CR7] Bogdanov, M., Ruff, C. C., & Schwabe, L. (2015). Transcranial Stimulation Over the Dorsolateral Prefrontal Cortex Increases the Impact of Past Expenses on Decision-Making. *Cerebral Cortex*, *December 2015*, bhv298. 10.1093/cercor/bhv298 10.1093/cercor/bhv298 26656728

[CR8] Bonaiuto JJ, de Berker A, Bestmann S (2016). Response repetition biases in human perceptual decisions are explained by activity decay in competitive attractor models. ELife.

[CR9] Brainard, D. H. (1997). The psychophysics toolbox. *Spatial Vision, 10*(4), 433–436. 9176952

[CR10] Braun A, Urai AE, Donner TH (2018). Adaptive history biases result from confidence-weighted accumulation of past choices. The Journal of Neuroscience.

[CR11] Camille N, Griffiths CA, Vo K, Fellows LK, Kable JW (2011). Ventromedial frontal lobe damage disrupts value maximization in humans. Journal of Neuroscience.

[CR12] Casula EP, Testa G, Bisiacchi PS, Montagnese S, Caregaro L, Amodio P, Schiff S (2017). Transcranial Direct Current Stimulation (tDCS) of the Anterior Prefrontal Cortex (aPFC) Modulates Reinforcement Learning and Decision-Making Under Uncertainty: a Double-Blind Crossover Study. Journal of Cognitive Enhancement.

[CR13] Chew T, Ho KA, Loo CK (2015). Inter- and intra-individual variability in response to transcranial direct current stimulation (tDCS) at varying current intensities. Brain Stimulation.

[CR14] Chib VS, Yun K, Takahashi H, Shimojo S (2013). Noninvasive remote activation of the ventral midbrain by transcranial direct current stimulation of prefrontal cortex. Translational Psychiatry.

[CR15] Cho RY, Nystrom LE, Brown ET, Jones AD, Braver TS, Holmes PJ, Cohen JD (2002). Mechanisms underlying dependencies of performance on stimulus history in a two-alternative forced-choice task. Cognitive, Affective, & Behavioral Neuroscience.

[CR16] Civai C, Miniussi C, Rumiati RI (2014). Medial prefrontal cortex reacts to unfairness if this damages the self: A tDCS study. Social Cognitive and Affective Neuroscience.

[CR17] Colzato LS, Sellaro R, van den Wildenberg WPM, Hommel B (2015). tDCS of medial prefrontal cortex does not enhance interpersonal trust. Journal of Psychophysiology.

[CR18] Faul F, Erdfelder E, Lang A-G, Buchner A (2007). G*Power 3: a flexible statistical power analysis program for the social, behavioral, and biomedical sciences. Behavior Research Methods.

[CR19] Fellows LK (2006). Deciding how to decide: Ventromedial frontal lobe damage affects information acquisition in multi-attribute decision making. Brain.

[CR20] Fregni F, Boggio PS, Nitsche M, Bermpohl F, Antal A, Feredoes E, Marcolin MA, Rigonatti SP, Silva MTA, Paulus W, Pascual-Leone A (2005). Anodal transcranial direct current stimulation of prefrontal cortex enhances working memory. Experimental Brain Research.

[CR21] Fründ I, Wichmann FA, Macke JH (2014). Quantifying the effect of intertrial dependence on perceptual decisions. Journal of Vision.

[CR22] Gandiga PC, Hummel FC, Cohen LG (2006). Transcranial DC stimulation (tDCS): A tool for double-blind sham-controlled clinical studies in brain stimulation. Clinical Neurophysiology.

[CR23] Gao J, Wong-Lin K, Holmes P, Simen P, Cohen JD (2009). Sequential effects in two-choice reaction time tasks: decomposition and synthesis of mechanisms. Neural Computation.

[CR24] Gläscher J, Adolphs R, Damasio H, Bechara A, Rudrauf D, Calamia M, Paul LK, Tranel D (2012). Lesion mapping of cognitive control and value-based decision making in the prefrontal cortex. Proceedings of the National Academy of Sciences of the United States of America.

[CR25] Greiner B (2015). Subject pool recruitment procedures: organizing experiments with ORSEE. Journal of the Economic Science Association.

[CR26] Hämmerer D, Bonaiuto JJ, Klein-Flügge M, Bikson M, Bestmann S (2016). Selective alteration of human value decisions with medial frontal tDCS is predicted by changes in attractor dynamics. Scientific Reports.

[CR27] Hare TA, Camerer CF, Rangel A (2009). Self-Control in Decision-Making Involves Modulation of the vmPFC Valuation System. Science.

[CR28] Ho KA, Taylor JL, Chew T, Gálvez V, Alonzo A, Bai S, Dokos S, Loo CK (2016). The Effect of Transcranial Direct Current Stimulation (tDCS) Electrode Size and Current Intensity on Motor Cortical Excitability: Evidence from Single and Repeated Sessions. Brain Stimulation.

[CR29] Hummel F, Celnik P, Giraux P, Floel A, Wu WH, Gerloff C, Cohen LG (2005). Effects of non-invasive cortical stimulation on skilled motor function in chronic stroke. Brain.

[CR30] Hummel F, Cohen LG (2005). Improvement of motor function with noninvasive cortical stimulation in a patient with chronic stroke. Neurorehabilitation and Neural Repair.

[CR31] Hunt LT, Kolling N, Soltani A, Woolrich MW, Rushworth MFS, Behrens TEJ (2012). Mechanisms underlying cortical activity during value-guided choice. Nature Neuroscience.

[CR32] Iyer MB, Mattu U, Grafman J, Lomarev M, Sato S, Wassermann EM (2005). Safety and cognitive effect of frontal DC brain polarization in healthy individuals. Neurology.

[CR33] JASP Team. (2019). *JASP (Version 0.11.1) [Computer software]*. https://jasp-stats.org/

[CR34] Jocham G, Hunt LT, Near J, Behrens TEJ (2012). A mechanism for value-guided choice based on the excitation- inhibition balance in prefrontal cortex. Nature Neuroscience.

[CR35] Kable JW, Glimcher PW (2007). The neural correlates of subjective value during intertemporal choice. Nature Neuroscienceeuroscience.

[CR36] Kidgell, D. J., Daly, R. M., Young, K., Lum, J., Tooley, G., Jaberzadeh, S., Zoghi, M., & Pearce, A. J. (2013). Different current intensities of anodal transcranial direct current stimulation do not differentially modulate motor cortex plasticity. *Neural Plasticity*. 10.1155/2013/603502 10.1155/2013/603502 PMC3614037 23577272

[CR37] Kuhl, J. (1994). Action versus state orientation: Psychometric properties of the Action Control Scale. ACS-90. In J. Kuhl & J. Beckmann (Eds.), *Volition and personality: Action versus state orientation* (pp. 47–59). Hogrefe.

[CR38] León, J. J., Sánchez-Kuhn, A., Fernández-Martín, P., Páez-Pérez, M. A., Thomas, C., Datta, A., Sánchez-Santed, F., & Flores, P. (2020). Transcranial direct current stimulation improves risky decision making in women but not in men: A sham-controlled study. *Behavioural Brain Research*, *382*(January). 10.1016/j.bbr.2020.112485 10.1016/j.bbr.2020.112485 31958518

[CR39] Liao C, Wu S, Luo YJ, Guan Q, Cui F (2018). Transcranial direct current stimulation of the medial prefrontal cortex modulates the propensity to help in costly helping behavior. Neuroscience Letters.

[CR40] Mainz V, Britz S, Forster SD, Drüke B, Gauggel S (2020). Transcranial Direct Current Stimulation of the Medial Prefrontal Cortex Has No Specific Effect on Self-referential Processes. Frontiers in Human Neuroscience.

[CR41] Manuel AL, Murray NWG, Piguet O (2019). Transcranial direct current stimulation (tDCS) over vmPFC modulates interactions between reward and emotion in delay discounting. Scientific Reports.

[CR42] Moos K, Vossel S, Weidner R, Sparing R, Fink GR (2012). Modulation of top-down control of visual attention by cathodal tDCS over right IPS. Journal of Neuroscience.

[CR43] Nguyen KP, Josić K, Kilpatrick ZP (2019). Optimizing sequential decisions in the drift–diffusion model. Journal of Mathematical Psychology.

[CR44] Nitsche MA, Cohen LG, Wassermann EM, Priori A, Lang N, Antal A, Paulus W, Hummel F, Boggio PS, Fregni F, Pascual-Leone A (2008). Transcranial direct current stimulation: State of the art 2008. Brain Stimulation.

[CR45] Nitsche MA, Paulus W (2001). Sustained excitability elevations induced by transcranial DC motor cortex stimulation in humans. Neurology.

[CR46] Nitsche MA, Paulus W (2011). Transcranial direct current stimulation - Update 2011. Restorative Neurology and Neuroscience.

[CR47] Padoa-Schioppa C (2013). Neuronal origins of choice variability in economic decisions. Neuron.

[CR48] Padoa-Schioppa C, Assad JA (2006). Neurons in Orbitofrontal Cortex Encode Economic Value. Nature.

[CR49] Padoa-Schioppa C, Assad JA (2008). The representation of economic value in the orbitofrontal cortex is invariant for changes of menu. Nature Neuroscience.

[CR50] Pelli, D. G. (1997). The VideoToolbox software for visual psycho- physics: transforming numbers into movies. *Spatial Vision, 10*(4), 437–442. 9176953

[CR51] Renard Y, Poulios K (2020). GetFEM: Automated FE Modeling of Multiphysics Problems Based on a Generic Weak Form Language. ACM Transactions on Mathematical Software.

[CR52] Rodewald K, Bartolovic M, Debelak R, Aschenbrenner S, Weisbrod M, Roesch-Ely D (2012). Eine normierungsstudie eines modifizierten trail making tests im Deutschsprachigen raum. Zeitschrift Fur Neuropsychologie.

[CR53] Rolls ET, McCabe C, Redoute J (2008). Expected value, reward outcome, and temporal difference error representations in a probabilistic decision task. Cerebral Cortex.

[CR54] Rushworth MFS, Noonan MAP, Boorman ED, Walton ME, Behrens TE (2011). Frontal Cortex and Reward-Guided Learning and Decision-Making. Neuron.

[CR55] Rustichini A, Padoa-Schioppa C (2015). A neuro-computational model of economic decisions. Journal of Neurophysiology.

[CR56] Saturnino GB, Siebner HR, Thielscher A, Madsen KH (2019). Accessibility of cortical regions to focal TES: Dependence on spatial position, safety, and practical constraints. NeuroImage.

[CR57] Scherbaum S, Dshemuchadse M, Leiberg S, Goschke T (2013). Harder than expected: Increased conflict in clearly disadvantageous delayed choices in a computer game. PLoS ONE.

[CR58] Scherbaum S, Frisch S, Leiberg S, Lade SJ, Goschke T, Dshemuchadse M (2016). Process dynamics in delay discounting decisions : An attractor dynamics approach. Judgement and Decision Making.

[CR59] Scherbaum S, Haber P, Morley K, Underhill D, Moustafa AA (2018). Biased and less sensitive: A gamified approach to delay discounting in heroin addiction. Journal of Clinical and Experimental Neuropsychology.

[CR60] Schoemann M, Scherbaum S (2020). From high- to one-dimensional dynamics of decision making: testing simplifications in attractor models. Cognitive Processing.

[CR61] Senftleben U, Schoemann M, Schwenke D, Richter S, Dshemuchadse M, Scherbaum S (2019). Choice perseveration in value-based decision making: The impact of inter-trial interval and mood. Acta Psychologica.

[CR62] Soetens E, Boer LC, Hueting JE (1985). Expectancy or automatic facilitation? Separating sequential effects in two-choice reaction time. Journal of Experimental Psychology: Human Perception and Performance.

[CR63] Soetens E, Melis A, Notebaert W (2004). Sequence learning and sequential effects. Psychological Research.

[CR64] Strait CE, Blanchard TC, Hayden BY (2014). Reward value comparison via mutual inhibition in ventromedial prefrontal cortex. Neuron.

[CR65] Teo F, Hoy KE, Daskalakis Z, Fitzgerald PB (2011). Investigating the role of current strength in tdcs modulation of working memory performance in healthy controls. Frontiers in Psychiatry.

[CR66] Thair H, Holloway AL, Newport R, Smith AD (2017). Transcranial direct current stimulation (tDCS): A Beginner’s guide for design and implementation. Frontiers in Neuroscience.

[CR67] Urai AE, de Gee JW, Tsetsos K, Donner TH (2019). Choice history biases subsequent evidence accumulation. ELife.

[CR68] Wallis JD, Miller EK (2003). Neuronal activity in primate dorsolateral and orbital prefrontal cortex during performance of a reward preference task. European Journal of Neuroscience.

[CR69] Wang M, Li J, Li D, Zhu C, Wang Y (2020). Modulation of income redistribution decisions by anodal tDCS over the medial prefrontal cortex. Neuroscience Letters.

[CR70] Yuan H, Tabarak S, Su W, Liu Y, Yu J, Lei X (2017). Transcranial direct current stimulation of the medial prefrontal cortex affects jsudgments of moral violations. Frontiers in Psychology.

[CR71] Zheng H, Huang D, Chen S, Wang S, Guo W, Luo J, Ye H, Chen Y (2016). Modulating the activity of ventromedial prefrontal cortex by anodal tDCS enhances the trustee’s repayment through altruism. Frontiers in Psychology.

