# Supplementary material for: Is value-based choice repetition susceptible to medial frontal transcranial direct current stimulation (tDCS)? A preregistered study
Source: Cogn Affect Behav Neurosci. 2021 Apr 2;21(4):747–62. doi: 10.3758/s13415-021-00889-7 (PMC8354960; doi:10.3758/s13415-021-00889-7)
Supplement: Supplementary file 1 — (PDF 375 kb) [file 13415_2021_889_MOESM1_ESM.pdf]

Supplementary Materials for

Is value-based choice repetition susceptible to medial frontal transcranial direct current stimulation (tDCS)? A preregistered study

Ulrike Senftleben[1*], Johanna Kruse[1], Franziska M. Korb[1], Stefan Goetz[2,3,4] & Stefan Scherbaum[1]

Author Note

This research was partly supported by the German Research Foundation (DFG) (grant SFB 940/2 2016, projects A6 and A8).

Affiliations

[1] Department of Psychology, Technische Universität Dresden, Germany
[2] University of Cambridge, School of Technology, Cambridge, CB2 1RX, UK
[3] Duke University, School of Medicine, Durham, NC 27710, USA
[4] Duke University, Pratt School of Engineering, NC 27708, USA

Author information

Ulrike Senftleben, ulrike.senftleben@tu-dresden.de, https://orcid.org/0000-0002-8403-0359;
Johanna Kruse, johanna.kruse@tu-dresden.de;
Franziska M. Korb, franziska.korb@tu-dresden.de, https://orcid.org/0000-0002-1005-0090;
Stefan Goetz, stefan.goetz@duke.edu, https://orcid.org/0000-0002-1944-0714;
Stefan Scherbaum, stefan.scherbaum@tu-dresden.de, https://orcid.org/0000-0002-4408-6016

*corresponding author

Measurement block

Estimation of indifference points

Indifference points describe the value difference of the small/near (SN) option and the large/far (LF) option where participants are indifferent, that is, the probability of choosing the SN option over the LF option is 50 %. Indifference points were estimated separately for each distance. For the estimation of the indifference points at a certain distance, we determined the point of inflection of a logistic function that was fitted to each participant's choice data (SN option vs. LF option) as a function of increasing value differences. This was carried out using the function logitfit from the StixBox mathematical toolbox by Anders Holtsberg (http://www.maths.lth.se/matstat/stixbox/). The fit was based on the model $\log[p/(1-p)] = b0 + X * b1$, where p is the probability that the choice is 1 (SN option) and not 0 (LF option), X represents value differences, and $b0$ and $b1$ represent the point estimates for the logistic function. In the experiment, indifference points were calculated after the measurement block in order to manipulate choices in the subsequent experimental block. For that calculation, we discarded the last ten trials of the measurement block because these trials might reflect time pressure more than they might reflect actual subjective values, and this was shown to improve the subsequent manipulation in our previous studies.

Behavioral results

Based on the indifference points estimated from the measurement block, the discounting parameter k can be obtained. This discounting parameter is typically used to describe discounting in intertemporal choice. It is obtained by fitting a hyperbolic model to the indifference points of each participant. This hyperbolic model assumes that participants prefer the LF option less and less the larger the delay to the LF option becomes (in our paradigm: the larger the distance to the LF option becomes). The parameter k represents how

steep this function is: a higher *k* corresponds to a higher devaluation of the delayed reward (i.e., stronger preference for SN choices, especially when the LF option is far away). Table S 1 gives an overview of the mean *k* values as well as other details on participants' behavior in the measurement block. Participants' individual discounting was highly correlated across sessions, $r = .66$, $p < .001$, see Figure S 1. Taken together, this indicates that 1) participants found the LF option less valuable the farther away it was (comparable to how people find monetary rewards less valuable the longer they have to wait for them), and that 2) this devaluation or discounting of the LF option was a stable preference across time.

Table S 1
Descriptive statistics for choices in the measurement block.

	Session 1	Session 2
Mean amount of trials (SD)	143.65 (32.27)	160.98 (31.89)
Mean amount of SN choices (SD)	95.15 (40.31)	97.73 (38.30)
Mean percentage of SN choices (SD)	63.47% (15.75%)	58.59% (13.75%)
Mean amount of LF choices (SD)	48.50 (13.65)	63.25 (14.23)
Mean percentage of LF choices (SD)	36.53% (15.75%)	41.41% (13.75%)
Mean *k*	.27 (.16)	.21 (.13)

SN = small/near, LF = large/far, SD = standard deviation

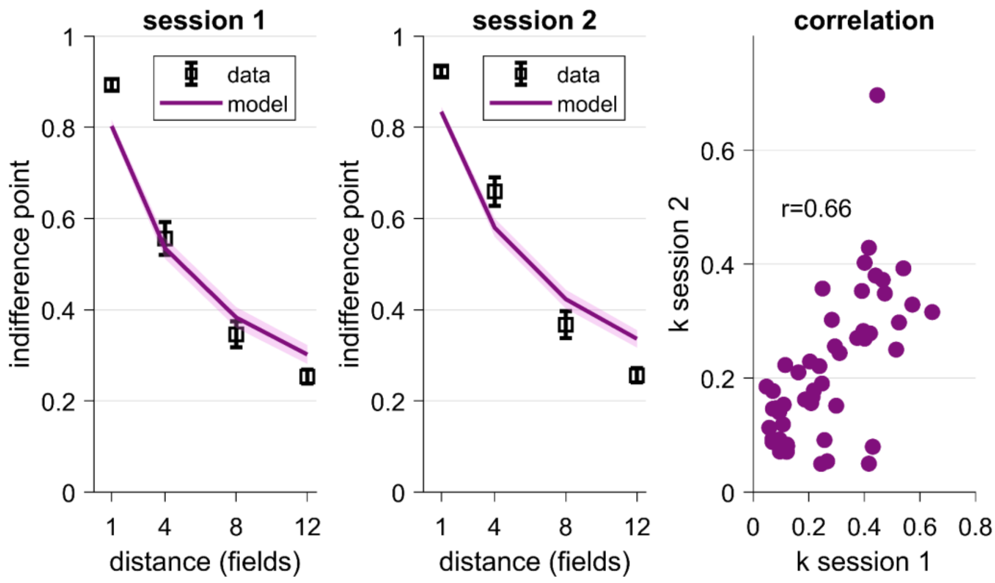

Figure S 1: Discounting across session. In the first two panels, data points are depicted in black and the hyperbolic model is depicted in purple. Error bars and error areas represent standard errors. The panel to the right shows a scatterplot of the Pearson correlation of the k parameter between the two sessions.

Experimental block

Table S 2
Mean decision times for repetitions and switches in target trials, only including trials under sham tDCS and where the bias trial manipulation was successful. Standard deviations in parentheses.

	Session 1	Session 2
Choice repetition	830.23 ms (139.86 ms)	769.88 ms (127.60 ms)
Choice switch	896.60 ms (169.46 ms)	808.57 ms (138.10 ms)

Manipulation check

In order to check that our manipulation of subjective value worked as intended, we looked at choices in the target trials (t) and in the directly preceding SN and LF bias trials. We expected that LF choice percentages should be high in LF bias trials, low in SN bias trials, and approximately 50% in neutral target trials. As Figure S 2 shows, our manipulation

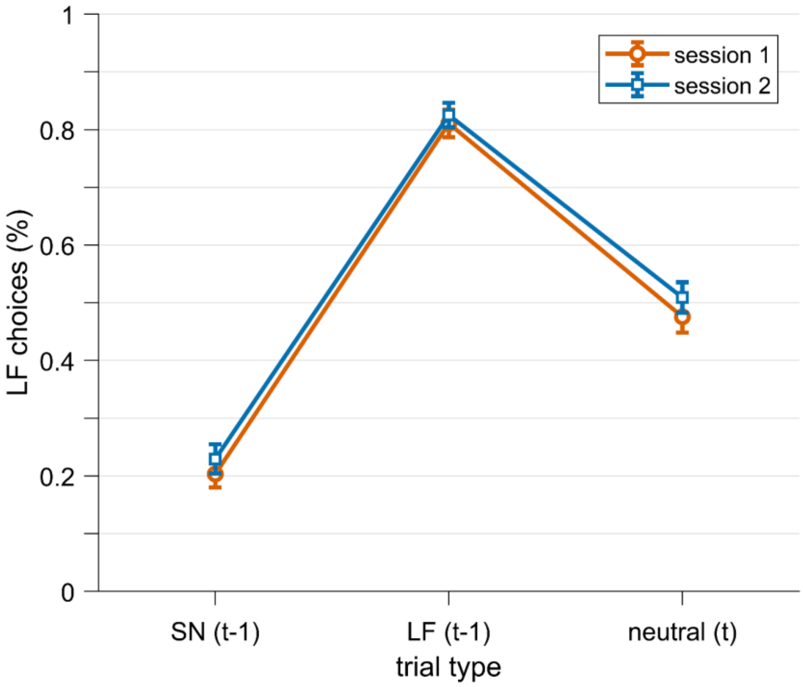

Figure S 2: Large/far (LF) choice percentages under sham tDCS, for small/near (SN) and LF bias trials directly preceding neutral target trials (t), for session 1 and session 2. Error bars represent standard errors.

successfully influenced choice behavior as expected in both sessions. This is supported by a repeated measures ANOVA with the factors trial type (SN, LF, target) and session on the percentage of LF choices: We found a significant main effect of trial type, $F(2,102) = 592.15$, $p < .001$, $\eta_p^2 = .91$, and no significant effects of session, $F(1,51) =1.15$, $p = .29$, $\eta_p^2 = .02$, or session x trial type, $F(2,102) = 0.26$, $p = .77$, , $\eta_p^2 = .005$. Post-hoc tests revealed significant differences between all three trial types, all *p*s < .001 (Holm-corrected).

Choice repetition: Single subject effects

Figure S 3 shows the repetition effect for each participant as indicated by the repetition index (percentage of LF choices after LF bias trials – percentage of LF choices after SN bias trials). In both sessions, the vast majority of participants (around 80%) showed a choice repetition bias as indicated by a positive repetition index. The magnitude of the repetition bias was

correlated across session, $r = .28$, $p = .04$, indicating that participants' repetition bias is at least somewhat stable across time.

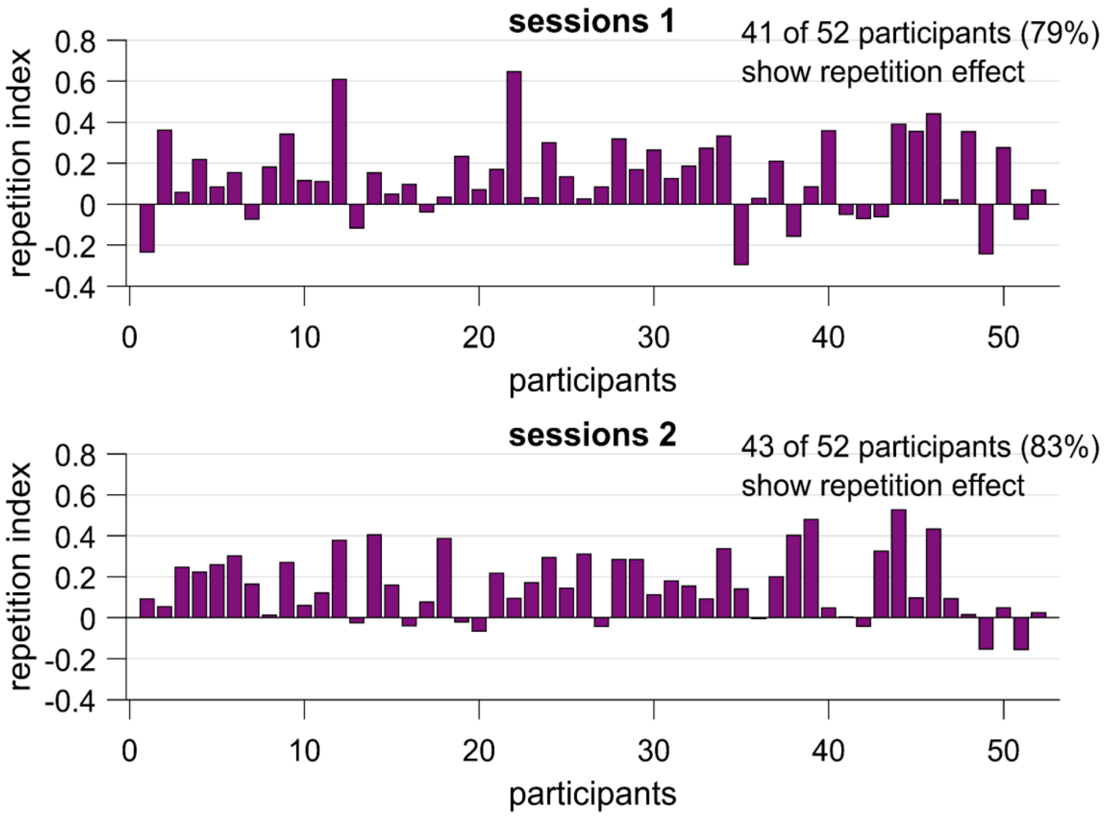

Figure S 3: Repetition index for single participants (sham condition), for each session. The repetition index is defined as the percentage of LF choices after LF bias trials minus the percentage of LF choices after SN bias trials (a positive value indicates choice repetition).

Bayesian analysis of tDCS effects

We ran Bayesian repeated measures ANOVAs for our confirmatory analyses of the effects of tDCS on choice repetition and on decision times. We used the default prior probabilities provided in JASP (multivariate Cauchly prior) and looked at the resulting Bayes factor BF_{01}, which indicates the likelihood of the data under the null hypothesis relative to the alternative hypothesis (i.e., $BF_{01} > 1$ indicates evidence in favor of the null hypothesis, $BF_{01} < 1$ indicates evidence in favor of the alternative hypothesis). The results for choice repetition are presented in Table S 3 and the results for decision times are presented in Table S 4. For both ANOVAs, the evidence favors the null hypothesis over the alternative hypothesis, hence further underlining our null effects findings

Table S 3
Results of the Bayesian repeated measures ANOVA with factor tDCS on the outcome choice repetition index (percentage of LF choices in LF-biased neutral targets minus percentage of LF choices in SN-biased neutral targets).

**Models**	**P(M)**	**P(M|data)**	**BF $_M$**	**BF $_{01}$**	**error %**
Null model	0.500	0.871	6.732	1.000	
tDCS	0.500	0.129	0.149	6.732	0.849

Note: P(M): prior probabilities; P(M|data): updated prior probabilities; BF_M: posterior model odds; BF_{01}: Bayes factor for H0 vs. H1.

Table S 4
Results of the Bayesian repeated measures ANOVA with factor tDCS on the outcome log-transformed decision times in all target trials.

**Models**	**P(M)**	**P(M|data)**	**BF $_M$**	**BF $_{01}$**	**error %**
Null model	0.500	0.924	12.147	1.000	
tDCS	0.500	0.076	0.082	12.147	0.908

Note: P(M): prior probabilities; P(M|data): updated prior probabilities; BF_M: posterior model odds; BF_{01}: Bayes factor for H0 vs. H1.

tDCS screening questionnaire

Our in-house questionnaire was in German (see English translation below). In case a participant answered any of the questions with “yes”, the experimenter contacted a physician at the Neuroimaging Center of the Technische Universität Dresden, Germany. The physician then evaluated the case and judged if the participant was cleared for tDCS or if the participant had to be excluded.

	**Yes**	**No**
Have you ever had a seizure?		
Has any of your relatives ever had a seizure?		
Have you ever experienced loss of consciousness or a syncope? If yes, please describe the incident.		
Have you ever had a head injury that was diagnosed as a concussion or that resulted in a loss of consciousness?		
Do you suffer from migraines?		
Do you have hearing problems or tinnitus?		
Do you have a cochlear implant?		
For female participants: Is there the possibility that you might be pregnant?		
Do you have any metal in your head such as shrapnel or surgical clips? If yes, please specify the material.		
Do you have any implanted brain stimulators (e.g., deep brain stimulator, epidural / subdural stimulator, vagus nerve stimulator)?		
Do you have a cardiac pacemaker or intracardial devices?		
Do you have a medical infusion device (e.g., insulin pump)?		
Are you currently taking any medications? If yes, please specify.		

Have you ever participated in a tDCS study before? If yes, did you experience any adverse reactions?		
Have you ever undergone an MRI scan? If yes, did you experience any problems?		
Are you currently suffering from a neurological or psychiatric condition (e.g., multiple sclerosis, stroke, dementia, depression, dementia)? If yes, please specify.		
Do you consume alcohol on a regular basis? If yes, how much?		
